# *PyMSQ*: a Python package for fast Mendelian sampling (co)variance and haplotype-based similarity in genomic selection

**DOI:** 10.1186/s12859-026-06392-5

**Published:** 2026-02-14

**Authors:** Abdulraheem Arome Musa, Norbert Reinsch

**Affiliations:** Research Institute for Farm Animal Biology (FBN), Wilhelm-Stahl-Allee 2, 18196 Dummerstorf, Germany

**Keywords:** Mendelian sampling variance, Gametic variance, Haplotype similarity matrices, Optimal mating decisions, Genetic diversity management

## Abstract

**Background:**

While genomic selection boosts rapid genetic gains by leveraging dense marker data for genomic estimated breeding values (GEBVs), prolonged application can reduce haplotype diversity and increase inbreeding. To address these risks, recent studies indicate that selecting on Mendelian sampling variance (MSV) and covariance (MSC) can partly mitigate the loss of genetic variation by exploiting within-family (co)segregation not captured by GEBVs alone. In parallel, theoretical advances have introduced a haplotype-based similarity measure that targets shared heterozygous segments, enabling more direct control over haplotype diversity, either standalone or in combination with conventional coancestry-based and genomic relationship matrices.

**Results:**

We present *PyMSQ*, an open-source Python package for estimating Mendelian sampling–related quantities that implements two key developments: (1) a matrix-based approach for computing MSV and MSC in single-trait, multi-trait, and zygotic contexts, and (2) a haplotype-based similarity matrix between parental Mendelian sampling terms. By combining this matrix-based framework with optimized scientific libraries, *PyMSQ* achieves up to 332-fold faster computations than *gamevar*, a publicly available alternative, while preserving numerical accuracy. Using a Holstein–Friesian dataset, we demonstrate *PyMSQ*’s effectiveness in deriving MSV and MSC, as well as its novel similarity measure, which complements standard genomic relationship matrices by explicitly quantifying shared heterozygous segments rather than overall allele sharing, thereby providing additional insights for balancing immediate gains with long-term diversity.

**Conclusion:**

By facilitating the practical use of MSV, MSC, and a haplotype-based similarity metric, *PyMSQ* enables breeders and quantitative geneticists to adopt haplotype diversity constraints, whether as a standalone criterion or in synergy with optimal contribution selection. This framework opens new possibilities for preserving key haplotypic segments, ultimately supporting more sustainable genomic selection strategies. *PyMSQ* is freely available under an MIT License at https://github.com/aromemusa/PyMSQ.

**Supplementary Information:**

The online version contains supplementary material available at 10.1186/s12859-026-06392-5.

## Background

Genomic selection relies on genomic prediction, which uses dense marker data and predictive models to estimate breeding values more accurately than traditional pedigree-based methods [1, 2]. Although selection on genomic estimated breeding values (GEBVs) has expedited short-term genetic progress, relying solely on GEBVs, without integrating additional criteria such as inbreeding, may exacerbate inbreeding and erode haplotype diversity over time [3–6]. Preserving genetic variation at the haplotype level is especially important for traits influenced by multiple linked loci, where the loss of crucial segments could irreversibly limit future breeding opportunities.

A key step toward more sustainable breeding decisions involves quantifying within-family genetic variation by way of Mendelian sampling variance (MSV) and covariance (MSC). These metrics require phased genotypes, a marker map, and estimated marker effects. Unlike GEBVs, an individual’s average genetic merit, MSV and MSC capture the range of possible gametic outcomes a parent (or mating pair) could produce [7–15]. Identifying parents whose gametes exhibit high variability for one or multiple traits can help breeders “hedge” against narrowing genetic diversity [14, 16–21]. MSV therefore fulfills two complementary roles. First, it supports short‑term gain: parents with larger MSV increase the chance that at least a few offspring will outrank their own GEBV, thereby raising the expected within‑family response (the rationale behind strategies such as “usefulness‑criterion” selection) [12, 13]. Second, it provides the variance component exploited by optimization Mendelian sampling-based optimal contribution selection [14, 20], coancestry‑based optimal contribution selection (OCS) [14], genomic‑mating [22], and optimal cross‑selection schemes [18], to balance short‑term gain with the long‑term preservation of diverse haplotypes. However, computing MSV and MSC at scale has historically been computationally challenging, especially for large populations with high-density marker panels. Early simulation-based methods that enumerate or randomly sample parental haplotypes [7–10] become impractical for tens or hundreds of thousands of markers.

Subsequent analytical methods express breeding values in terms of marker effects [11, 23, 24], yet many still require constructing a parent-specific (co)variance matrix with phase indicators for heterozygous loci, an intensive step in large populations with dense marker sets. In contrast, Musa and Reinsch [14] recently proposed an efficient matrix-based representation that sets up parent-specific marker effects (including phase indicators for heterozygous loci) while building only a single population covariance matrix for each chromosome. This approach avoids creating a unique covariance matrix per parent, thereby enabling rapid computation of MSV and MSC under single-trait, multi-trait, and zygotic models. Beyond speed, the same framework yields a haplotype-based similarity matrix between parental Mendelian sampling terms that complements coancestry or genomic relationship matrices (GRMs) by targeting shared heterozygous segments (see “Haplotype‑Based Similarity” below). This measure quantifies the extent to which MSVs of potential parents arise from identical chromosomal segments. By optimizing mate selection to minimize parental similarities, breeders can preserve broader allelic diversity while maintaining expected genetic gain [14, 20].

Despite this theoretical progress, accessible software implementing both the matrix-based MSV/MSC framework and haplotype-based similarity has been limited. Many breeding programs still rely on simulation-heavy or inefficient analytical methods (e.g., *gamevar* [24]) that do not provide haplotype-level diversity metrics or that repeat computationally demanding steps for each parent. To bridge this gap, we developed *PyMSQ*, an open-source Python package for estimating Mendelian sampling–related quantities (MSQ) that merges the efficient MSV/MSC derivations of Musa and Reinsch [14] with haplotype-based similarity into a single platform. By leveraging optimized scientific libraries, *PyMSQ* can handle large datasets, multi-trait analyses, and zygotic variance calculations more feasibly than older methods.

In what follows, we detail *PyMSQ*’s design and implementation, validate its performance on a Holstein–Friesian dataset, and demonstrate how breeders can integrate MSV, MSC, and haplotype-based similarity to maintain diversity while achieving robust genetic gains in modern genomic selection programs.

## Implementation

### Overview

*PyMSQ* is a Python 3.8 + package designed to compute MSV and MSC, alongside a haplotype-based similarity metric, using the matrix-based framework presented by Musa and Reinsch [14]. By uniting parent-specific phase indicators with a single population covariance matrix ${\mathbf{R}}^{c}$ per chromosome, *PyMSQ* eliminates the need for parent-specific covariance matrices, thus significantly shortening runtime. Built primarily with *NumPy* and *pandas* for data manipulation, it also uses *Numba* to just-in-time (JIT) compile numeric routines, enabling faster processing of large genomic datasets.

Although written in Python, *PyMSQ* is compatible with R via the *reticulate* package, ensuring straightforward integration with R-centric breeding pipelines. The following subsections describe *PyMSQ*’s internal data structures, matrix computations, and core algorithms. Comprehensive package documentation, including examples and user guides, is available at https://github.com/aromemusa/PyMSQ.

### Data inputs and preparation

All genotype data must be fully phased and free of missing calls; *PyMSQ* neither performs phasing nor imputation. Users should provide genotype arrays (or DataFrames) in which each marker is assigned one of the two parental haplotypes. A genetic map is also required, specifying a chromosome identifier (e.g., 1, 2) for each marker, along with either a genetic distance ($d_{kl}^{c}$) or recombination rate ($r_{kl}^{c}$) between markers $k$ and $l$ on a chromosome $c$. These values populate the population-level covariance matrix ${\mathbf{R}}^{c}$ for each chromosome $c$, reflecting linkage disequilibrium patterns [14]. Markers on different chromosomes are considered independent.

Finally, marker-effect estimates must be supplied (for example, SNP effects from a genomic prediction model such as SNP best linear unbiased prediction (SNP-BLUP) or from genomic BLUP (GBLUP) by back-solving), ensuring their order and dimension exactly match the SNP order of the phased genotype data. In a single-trait setting, *PyMSQ* expects a vector ${\mathbf{m}}^{c} \in {\mathbb{R}}^{L}$ for each chromosome $c$, where $L$ denotes the number of SNP markers on chromosome $c$. For multiple traits, it requires a matrix ${\mathbf{M}}^{c} \in {\mathbb{R}}^{T \times L}$, where $T$ is the number of traits. *PyMSQ* terminates if it detects inconsistencies in marker order or shape among these datasets, prompting users to correct input files.

### Construction of the population covariance matrix

Musa and Reinsch [14] emphasize constructing a single population covariance matrix ${\mathbf{R}}^{c} \in {\mathbb{R}}^{L \times L}$ per chromosome $c$, thereby avoiding parent-specific covariance overhead. The off-diagonal entries $\rho_{kl}^{c}$ encode the expected recombination-adjusted linkage between markers $k$ and $l$. *PyMSQ* supports two mapping functions:

1.1. Haldane’s function (assuming no interference) [11, 25]:

$$\rho_{kl}^{c} = \frac{{\exp ( - 2d_{kl}^{c} )}}{4}$$ or $$\frac{{(1 - 2r_{kl}^{c} )}}{4}$$.

2.2. Linear approximation (with $\rho_{kl}^{c} = 0$ if $r_{kl}^{c} \ge 0.5$ (or $d_{kl}^{c} \ge 0.5$ Morgans) [23, 24]:

$$\rho_{kl}^{c} = \left( { - \frac{{d_{kl}^{c} }}{2} + 0.25} \right)$$ or equivalently $$\left( { - \frac{{r_{kl}^{c} }}{2} + 0.25} \right)$$.

In both models, the diagonal elements of ${\mathbf{R}}^{c}$ are 0.25 [11, 14], reflecting the variance contribution of a fully heterozygous locus. Internally, *PyMSQ* stores each ${\mathbf{R}}^{c}$ in a dictionary or list for quick access.

### Parent-specific marker effects

Where earlier methods often built a phase-adjusted matrix ${\mathbf{R}}_{i}^{c}$ for each parent $i$ [11, 23, 24], *PyMSQ* encodes phase indicators directly into marker-effect vectors (or matrices), following Musa and Reinsch [14]. Let ${\mathbf{m}}^{c} \in {\mathbb{R}}^{L}$ be the additive marker-effect vector for chromosome $c$. For parent $i$, *PyMSQ* constructs a vector ${\mathbf{m}}_{i}^{c}$ by multiplying each entry in ${\mathbf{m}}^{c}$ by a phase indicator $\delta_{ik}$:

$$ m_{ik}^{c} = \delta_{ik} m_{k}^{c} ,{\text{ with }}\delta_{ik} = \left\{ {\begin{array}{*{20}c} { + 1} \\ { - 1} \\ 0 \\ \end{array} } \right. \, \begin{array}{*{20}c} {{\mathrm{if}}\;{\text{ parent}}\; \, i\;{\text{ has}}\;{\text{ the}}\;{\text{ reference}}\;{\text{ allele }}\;{\text{on }}\;{\mathrm{the}}\;{\text{ first}}\;{\text{ haplotype }}\;{\mathrm{at}}\;{\text{ marker}}\; \, k} \\ {{\text{if }}\;{\mathrm{parent}}\; \, i\;{\text{ has}}\;{\text{ that}}\;{\text{ allele}}\;{\text{ on}}\;{\text{ the}}\;{\text{ second }}\;{\mathrm{haplotype}}\;{\text{ at}}\;{\text{ marker}}\; \, k \, } \\ {{\mathrm{if}}\;{\text{ parent }}\;i \, \;{\text{is }}\;{\mathrm{homozygous}}\;{\text{ at}}\;{\text{ marker}}\; \, k{. }} \\ \end{array} $$

This marker-effect adjustment eliminates the need for parent-specific covariance matrices and smoothly extends to multi-trait contexts by applying $\delta_{ik}$ to each rows of ${\mathbf{M}}^{c} \in {\mathbb{R}}^{T \times L}$.

### Computing MSV and MSC for gametes

Once ${\mathbf{R}}^{c}$ and ${\mathbf{m}}_{i}^{c}$ are established, the MSV of parent $i$ (single trait) becomes:

1 $$ \sigma_{{b_{i} }}^{2} = \sum\limits_{c} {({\boldsymbol{m}}_{i}^{c} )^{\prime } {\boldsymbol{R}}^{c} {\boldsymbol{m}}_{i}^{c} } , $$

while in multi-trait settings, each chromosome’s marker-effect matrix ${\mathbf{M}}_{i}^{c}$ yields:

2 $$ {\mathbf{V}}_{i} = \sum\limits_{c} {{\mathbf{M}}_{i}^{c} {\mathbf{R}}^{c} \left( {{\mathbf{M}}_{i}^{c} } \right)^{\prime } } , $$

where ${\mathbf{V}}_{i}$ contains MSVs on the diagonal and MSCs off-diagonal [14]. The variance of the aggregate genotype is computed via ${\mathbf{a^{\prime}V}}_{i} {\mathbf{a}}$, given a vector of index weight ${\mathbf{a}} \in {\mathbb{R}}^{T \times 1}$.

### Computing MSV and MSC for zygotes (parent-pair)

When considering a parent pair $ij$, *PyMSQ* sums each parent’s gametic variance to derive the (co)variance of their potential offspring [11]:

3 $$ var\left( {{\mathbf{b}}_{ij} } \right) = \sum\limits_{c} {\left[ {{\mathbf{M}}_{i}^{c} {\mathbf{R}}^{c} \left( {{\mathbf{M}}_{i}^{c} } \right)^{\prime } + {\mathbf{M}}_{j}^{c} {\mathbf{R}}^{c} \left( {{\mathbf{M}}_{j}^{c} } \right)^{\prime } } \right]} = {\mathbf{V}}_{ij} . $$

In single-trait mode, this reduces to $\sigma_{{b_{ij} }}^{2}$. This enables direct comparisons of within-family (co)variation for multiple mating pairs under single- or multi-trait models. The aggregated genotype variance for pair $ij$ is ${\mathbf{a^{\prime}V}}_{ij} {\mathbf{a}}$, and *PyMSQ* outputs each trait’s MSV, the aggregate MSV, and any inter-trait covariances.

### Haplotype-based similarity

*PyMSQ* implements the haplotype-based similarity measure from Musa and Reinsch [14], defined as a similarity matrix between the Mendelian sampling terms of different parents, explicitly targeting shared heterozygous segments among parents. Specifically, the similarity measure quantifies the expected covariance between the additive genetic values of gametes (Mendelian sampling terms) produced by pairs of parents. In single-trait scenarios, the similarity $s_{ij}$ between parents $i$ and $j$ is:

4 $$ s_{i,j} = \sum\limits_{c} {\left| {\left( {{\mathbf{m}}_{i}^{c} } \right)^{\prime } {\mathbf{R}}^{c} {\mathbf{m}}_{j}^{c} } \right|} , $$

where the absolute value maintains invariance to haplotype order. A similarity matrix ${\mathbf{S}} \in {\mathbb{R}}^{N \times N}$ for $N$ parents can be set up such that a parent’s self-similarity equals its MSV, i.e., $s_{i,i} = \sigma_{{b_{i} }}^{2}$. Marker-effect values in ${\mathbf{m}}_{i}^{c}$ and ${\mathbf{m}}_{j}^{c}$ are set to zero for loci homozygous in both parents, as these loci do not generate MSV and hence do not contribute to similarity. Consequently, high similarity values identify parent pairs whose MSVs originate from the same chromosomal segments, whereas low similarity values indicate parents with MSVs arising from distinct chromosomal regions. Breeding schemes can thus optimize mate selection by minimizing average similarity $s_{ij}$, effectively preserving allelic diversity and moderating long-term inbreeding without compromising immediate genetic gain.

For multi-trait contexts, a user-specified index-weight vector ${\mathbf{a}}$ yields:

$$ s_{ij} = \sum\limits_{c} {\left| {{\mathbf{a^{\prime}}} \cdot \left( {{\mathbf{M}}_{i}^{c} {\mathbf{R}}^{c} \left( {{\mathbf{M}}_{j}^{c} } \right)^{\prime } } \right) \cdot {\mathbf{a}}} \right|} . $$

*PyMSQ* also calculates similarities $s_{ij,uv}$ between zygotes from parent pairs $ij$ and $uv$:

5 $$ s_{ij,uv} = \sum\limits_{c} {\left[ {\left| {{\mathbf{a^{\prime}}} \cdot \left( {{\mathbf{M}}_{i}^{c} {\mathbf{R}}^{c} \left( {{\mathbf{M}}_{u}^{c} } \right)^{\prime } } \right) \cdot {\mathbf{a}}} \right| + \left| {{\mathbf{a^{\prime}}} \cdot {\mathbf{M}}_{j}^{c} {\mathbf{R}}^{c} \left( {{\mathbf{M}}_{v}^{c} } \right)^{\prime } \cdot {\mathbf{a}}} \right|} \right]} , $$

placing the respective MSVs on the diagonal [14]. Here, the row vectors in ${\mathbf{M}}_{u}^{c}$ and ${\mathbf{M}}_{v}^{c}$ correspond to the trait-specific marker effects of parents $u$ and $v$ for chromosome $c$.

### Standardized similarity

Because similarity values can range widely, *PyMSQ* offers a standardized matrix ${\mathbf{K}}$ derived as:

6 $$ {\mathbf{K}} = {\mathbf{D}}^{ - 1} \cdot {\mathbf{S}} \cdot {\mathbf{D}}^{ - 1} , $$

where ${\mathbf{D}}$ is diagonal, holding each parent’s Mendelian standard deviation $\sqrt {\sigma_{{b_{i} }}^{2} }$. This transforms off-diagonal entries to $\left[ {0,1} \right]$ and can help breeders impose constraints on haplotype similarity [14, 20].

### Selection criteria

Following Musa and Reinsch [14], let $b_{i}$ be the GEBV of parent $i$, and $\sigma_{{b_{i} }}^{2}$ its MSV as defined in Eq. (1). In the single-trait setting, *PyMSQ* computes $b_{i} = \sum\limits_{c = 1}^{C} {\left( {{\mathbf{c}}_{i}^{c} } \right)^{\prime } {\mathbf{m}}^{c} }$, where ${\mathbf{c}}_{i}^{c} \in {\mathbb{R}}^{L}$ is the marker genotype vector of parent $i$ on chromosome $c$, with entries coded as 1, 0, and − 1 for homozygous reference, heterozygous, and homozygous alternative genotypes, respectively. The corresponding single-trait marker-effect vector is ${\mathbf{m}}^{c} \in {\mathbb{R}}^{L}$, obtained from genomic prediction models [1]. In multi-trait applications, $b_{i}$ is interpreted as the value of a linear index of traits (with user-specified index weights), and the associated MSV is obtained as stated in Eq. (2) for that index. It then derives an index $I_{i} = b_{i} + \lambda \sigma_{{b_{i} }}$, combining GEBV and MSV, where $\sigma_{{b_{i} }}$ represents the Mendelian standard deviation. The constant $\lambda$ may reflect selection intensity [13] or optimize the probability of producing top-ranking offspring (e.g., $\sqrt 2 x$, where $x$ is the standardized normal truncation point of the selected proportion [12]). Under a zygotic approach, GEBVs and indices for a parent pair $ij$ are averaged to explore the joint potential.

### Algorithmic and software optimization

Most core operations in *PyMSQ*, such as setting up ${\mathbf{R}}^{c}$, ${\mathbf{m}}_{i}^{c}$ or ${\mathbf{M}}_{i}^{c}$, are vectorized or just-in-time (JIT) compiled using *Numba*, reducing Python’s overhead in loops and arithmetic on large arrays. For instance, repetitive tasks like $({\mathbf{m}}_{i}^{c} )^{\prime}{\mathbf{R}}^{c} {\mathbf{m}}_{i}^{c}$ benefit significantly from JIT speedups. The software caches each parent’s phase-adjusted marker effects to avoid recomputing ${\mathbf{m}}_{i}^{c}$ or ${\mathbf{M}}_{i}^{c}$ multiple times. It also parallelizes matrix multiplications, leveraging *NumPy*’s underlying BLAS routines (gemv/gemm).

### Outputs and integration

*PyMSQ* returns results primarily as *NumPy* arrays or *pandas* DataFrames, including MSV, MSC, and haplotype-based similarity matrices, facilitating export to.csv or.npy. Because many breeding analyses are R-based, *PyMSQ* is fully callable from R via *reticulate*, supporting seamless integration with other R-based genomic pipelines (e.g., *rrBLUP*, *BGLR*). By accommodating both Python and R environments, *PyMSQ* aims to reach a broad community of breeders and geneticists.

In large, centralized livestock evaluation systems (for example, national dairy or beef cattle evaluations), we envision that MSV, MSC, and haplotype-based similarity matrices will typically be computed centrally at evaluation centers or large-scale breeding units where phased genotypes and genomic predictions are available. These pre-computed values can then be securely and efficiently distributed (e.g., via routine evaluation reports or secure APIs) to breeding advisors or farms. Thus, direct access to raw genotype data at the farm level is unnecessary. Furthermore, *PyMSQ* readily accommodates complex multi-trait indices involving numerous traits or subindices, since multi-trait indices are easily represented by single weighted marker-effect vectors, ensuring computational efficiency. In such settings, MSV/MSC can be combined with GEBVs to construct usefulness-criterion and related within-family indices [12, 13, 19, 23, 26], and the resulting haplotype-based similarity (or its standardized form ${\mathbf{K}}$) can be passed as a diversity kernel to existing optimal-contribution or genomic-mating solvers to obtain diversity-aware assortative mating plans in artificial insemination and multiple ovulation and embryo transfer schemes (see also Musa and Reinsch [14]).

In closed or company-run breeding programs where a single organization controls genotyping, genomic prediction, and mating decisions (e.g., private crop and horticultural breeding programs, commercial aquaculture schemes, or nucleus poultry and pig populations), *PyMSQ* can be integrated in an analogous way. In these settings, marker effects from the in-house prediction models are combined with phased genotypes and the genetic map to derive MSV, MSC, and haplotype-based similarity matrices for the candidate parents, which can then be supplied directly to existing optimal-contribution, cross-selection, or mating-optimization tools in those programs to implement diversity-aware selection and mating decisions.

## Data and analysis

Using *PyMSQ* and a publicly available Holstein–Friesian cattle dataset [14], we derived MSCs and haplotype similarity matrices for the aggregate genotype of gametes, assigning equal index weights of 1. The dataset includes 265 cows from five paternal half-sib families (sizes 32–106), along with a physical map, genotypic data, pedigree information, and phenotypes for three milk traits, pH, fat yield (FY), and protein yield (PY) [27, 28]. Marker effects for this population had been estimated previously [27], and the genotypes were phased into haplotypes (yielding 10,304 markers). Approximate genetic map positions were also established [14]. These data files (map, phased genotypes, marker effects, and pedigree details) are bundled with *PyMSQ* to ensure full reproducibility.

Following the computation of MSVs and MSCs in *PyMSQ*, we visualized the results with *ggplot2* (v 3.3.3) [29], generating density plots of MSVs and MSCs. We then generated and examined haplotype similarity matrices with *corrplot* (v 0.92) [30] to assess interrelationships among parental genotypes. Supporting scripts and their outputs are included in Additional File 1.

To compare *PyMSQ*’s performance with *gamevar* [24], we ran simulation studies with two single-chromosome scenarios, each with 10 traits. In the Individuals scenario, we fixed the number of markers and varied the number of individuals from 5000 to 100,000 to study scaling in population size. In the Markers scenario, we fixed the number of individuals at 500 and varied the number of SNPs on a single chromosome from 2500 to 50,000 to isolate scaling in per-chromosome marker density; the lower part of this range (2500–5000 SNPs) is a realistic upper bound for per-chromosome densities on commonly used ~ 50 k bovine chips, whereas the upper part (20,000–50,000 SNPs per chromosome) corresponds to densities encountered on high- and ultra-high-density panels and thus represents a demanding per-chromosome stress test. For these Individuals and Markers scenarios, each dataset underwent 10 replicates, measuring the computation time (minutes) and peak memory usage (GB) needed to compute MSCs, as well as the time and memory required for haplotype similarity matrices. To complement these single-chromosome stress tests with a more conventional whole-genome setting, we constructed an additional benchmark based on the bovine 50 k marker map of Hampel et al. [28], comprising 39,780 autosomal SNPs distributed over 29 chromosomes. Using this map, we simulated phased haplotypes and marker effects for a single trait and for 10 traits and used *PyMSQ* to compute MSV/MSC and haplotype-based similarity for sets of candidate parents up to 100,000 individuals, recording computation time and peak memory usage for each parent-set size. All computations were conducted on a multi-user Linux server (Intel Xeon Gold 6130, 128 CPU cores at 2.10 GHz, 1536 GB RAM). To avoid oversubscription on the shared node and to obtain stable runtimes across replicates, we capped BLAS/OpenMP parallelism for *PyMSQ* at 32 threads. *gamevar* was run in its standard configuration as provided by the authors (single-threaded). Detailed scripts and outputs are provided in Additional File 1.

## Results

### Mendelian variances and trait correlations

**Fig. 1:**
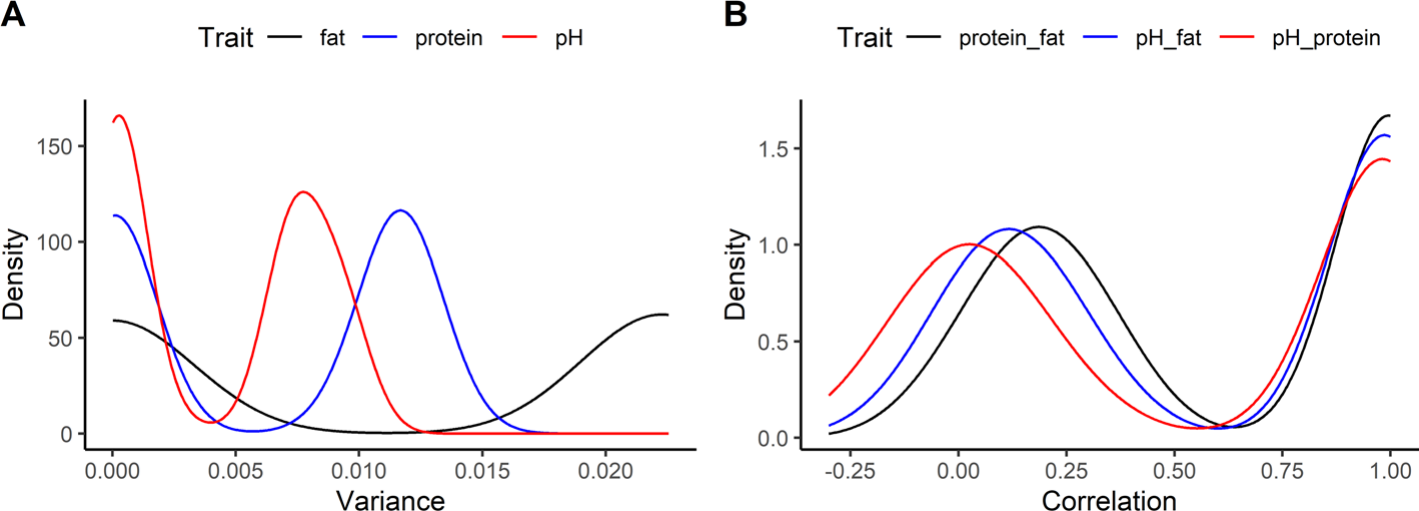
Density plots of Mendelian sampling variances and trait correlations. Panel A displays the variance in fat yield (kg), protein yield (kg), and pH (mol/L). Panel B shows correlations between these traits

Applying *PyMSQ* to the Holstein–Friesian dataset revealed a wide range of MSVs for the three milk traits: pH, FY, and PY. As illustrated in Fig. 1A, MSV distributions for each trait showed coefficients of variation exceeding 90%, underscoring the substantial within-family genetic diversity stemming from heterozygous loci and linkage phase [11, 14]. Each density curve is clearly bimodal, with a mass of cows at very low MSV and a second mode at higher MSV. A natural interpretation is that cows in the low-MSV mode are largely homozygous at segments with moderate-to-large effects on the trait, or carry opposing-effect haplotypes in repulsion phase, so that segregation generates little MSV. Cows in the high-MSV mode remain heterozygous at one or more haplotypes with sizeable effects, typically in coupling phase across closely linked loci, which inflates MSV. For FY, for example, the separation between the low- and high-MSV modes matches what would be expected when a large-effect quantitative trait locus such as *DGAT1* segregates in the population, as reported in previous Holstein studies [8, 11, 23, 31], superimposed on many smaller-effect contributors.

Moving beyond single-trait analyses, we derived multi-trait MSCs for each cow, thus quantifying how alleles for pH, FY, and PY co-segregate. The resulting Mendelian correlations between traits (Fig. 1B) are dominated by moderately to strongly positive values, with a smaller subset of cows showing clearly negative correlations and comparatively few observations in the intermediate range around 0.5–0.6. This pattern suggests that, conditional on the realized haplotypes, most cows either carry major-effect segments whose marker-effect signs are largely concordant across traits (yielding high positive correlations) or combinations of pleiotropic and linked loci with partially antagonistic effects (yielding near-zero or negative correlations) [11, 23, 32], whereas realized covariance structures that generate Mendelian correlations around 0.5–0.6 are relatively rare in this dataset. These findings highlight opportunities for breeders to strategically select or mate parents to enhance beneficial multi-trait associations or reduce antagonistic linkages.

### Haplotype-based similarity and its standardization

**Fig. 2:**
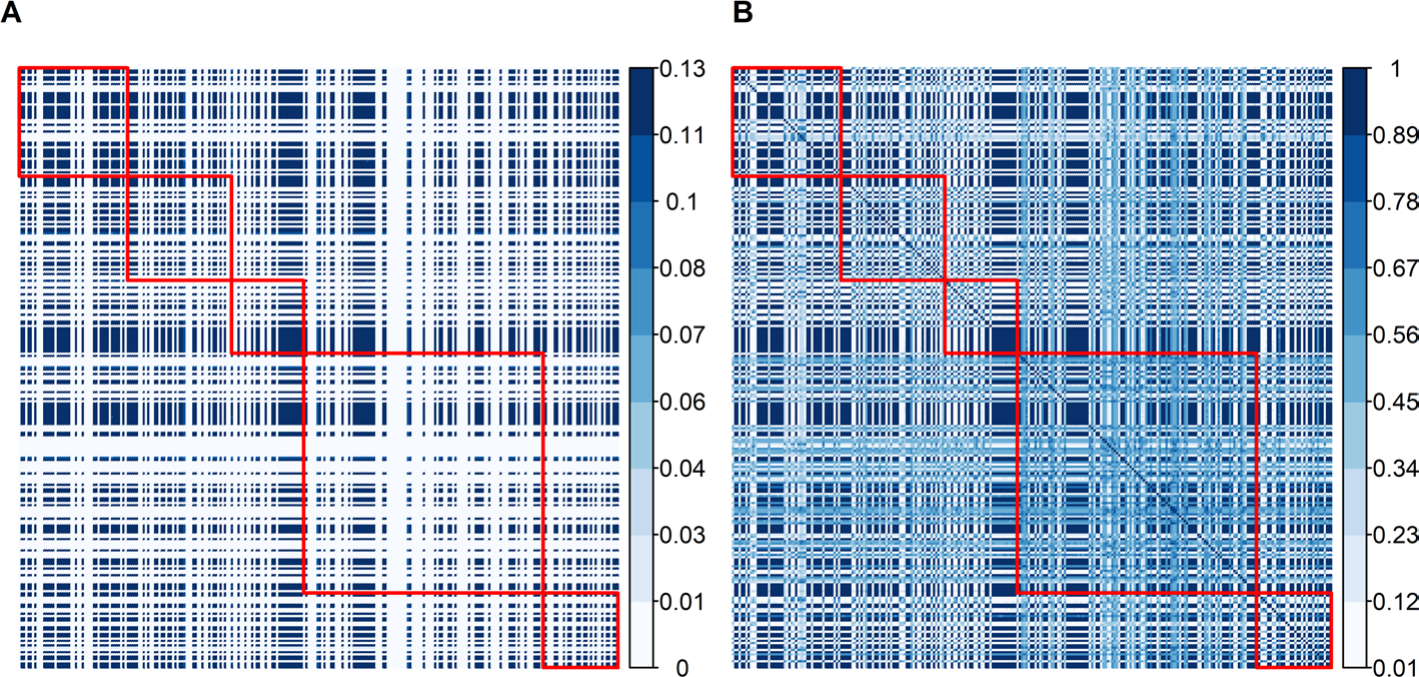
Unstandardized (**A**) and standardized (**B**) similarity matrices for the aggregate genotype of 265 Holstein–Friesian cows from five half-sib families (delineated by red lines). In (A), diagonal values represent individual Mendelian sampling variances, and off-diagonal values represent similarity between parent pairs in terms of shared gametic variance. Panel (**B**) shows the standardized similarity matrix, where values range from 0 (no similarity) to 1 (maximum similarity)

We next constructed a haplotype-based similarity matrix for an aggregate genotype combining pH, FY, and PY with equal weights. Diagonal elements in this matrix correspond to each cow’s MSV, and off-diagonals measure the overlap in high-impact heterozygous segments [14]. Figure 2A illustrates that some pairs of cows share extensive haplotype regions, while others remain relatively dissimilar. We also generated a standardized matrix ${\mathbf{K}}$ (Fig. 2B) by dividing off-diagonal entries by the product of each parent’s Mendelian standard deviation, which rescales similarities to a [0,1] interval and accentuates smaller overlaps. This haplotype-level focus complements the GRM by highlighting heterozygous loci relevant to within-family variance, rather than just overall allele sharing [14, 20]. In practical breeding settings, imposing limits on pairwise similarity can retain critical haplotypes, thus promoting the transmission of diverse haplotypes, thereby moderating rather than accelerating long-term inbreeding.

### Comparative benchmarking with gamevar

**Fig. 3:**
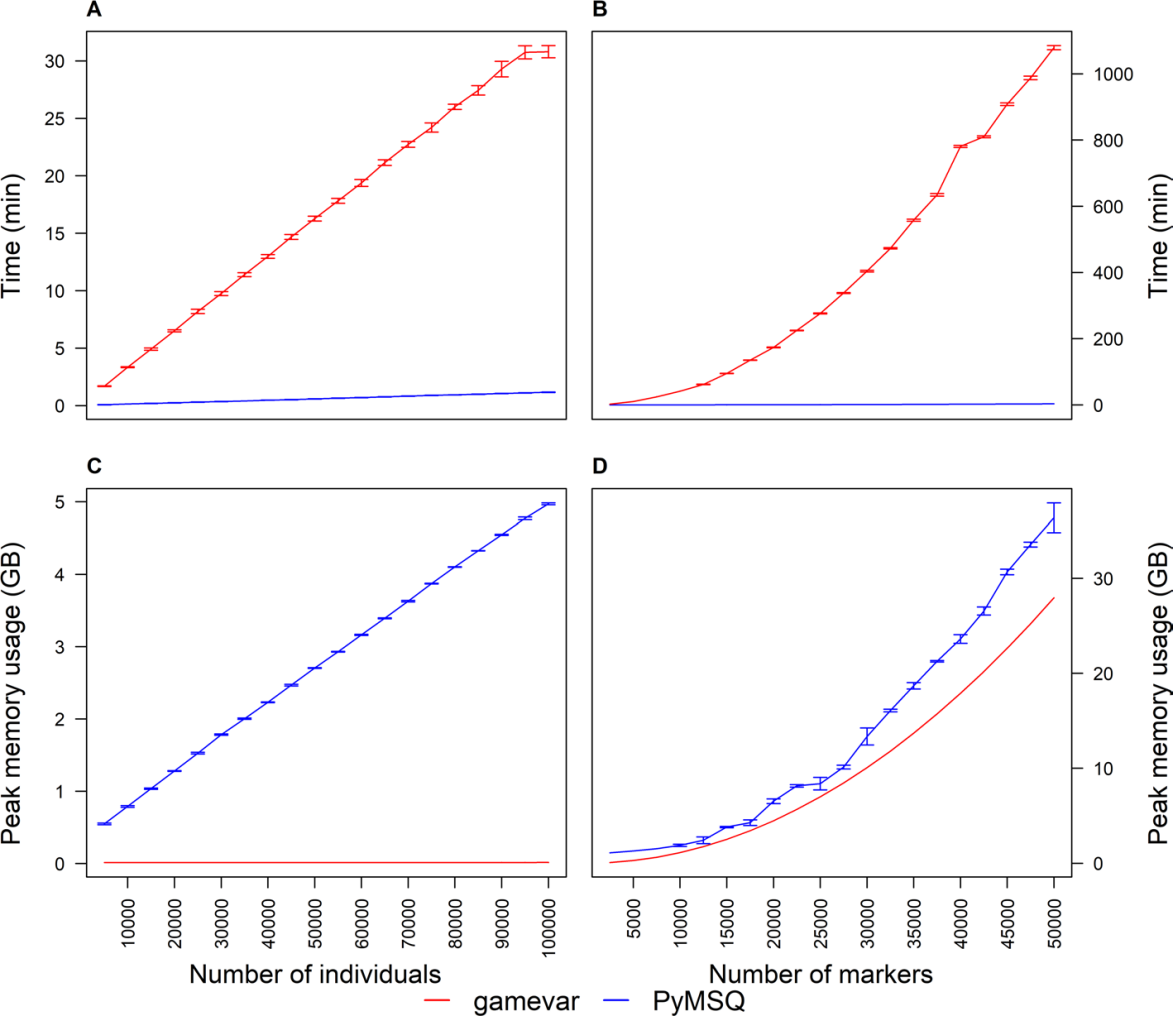
Benchmark plots comparing computation time (Panels **A**, **B**) and memory usage (Panels **C**, **D**) for *PyMSQ* and *gamevar*. In the Individuals scenario (Panels **A**, **C**), MSV/MSC are computed on a single chromosome with 1000 markers while the number of individuals increases from 5000 to 100,000. In the Markers scenario (Panels **B**, **D**), MSV/MSC are again computed on a single chromosome, with the number of individuals fixed at 500 and the number of markers increasing from 2500 to 50,000

In the Individuals scenario, the number of markers was fixed at 1000 while individual counts ranged from 5000 to 100,000. *PyMSQ*’s runtime (Fig. 3A) increased from about 0.07–1.16 min, whereas *gamevar* required 1.69–30.80 min, yielding a time ratio (*gamevar*/*PyMSQ*) of 23 to 27. Memory usage (Fig. 3C) showed the opposite trend: *PyMSQ* scaled from ~ 0.55 to ~ 4.97 GB, while *gamevar* remained near 0.015 GB throughout, reflecting *gamevar*’s genotype line-streaming approach versus *PyMSQ*’s in-memory design.

In the Markers scenario (Fig. 3B, D), 500 individuals were held constant while marker counts rose from 2500 to 50,000. *PyMSQ*’s runtime climbed from 0.03 to 3.25 min, whereas *gamevar* soared from 2.44 to ~ 1078.89 min, producing time ratios of up to 332. *Gamevar*’s memory usage grew from ~ 0.07 to ~ 27.95 GB, reflecting the quadratic expansion of marker-level arrays. Although *PyMSQ*’s memory usage also increased (from ~ 1.10 to ~ 36.35 GB), its matrix-based approach remained markedly faster, particularly for higher marker densities and multi-trait analyses. Overall, *PyMSQ* provides substantial speed advantages over *gamevar*, provided sufficient RAM is available.

In the additional 50 k-chip multi-chromosome benchmark described in the Data and analysis section, MSV/MSC runtimes for single-trait indices increased from about 0.13 min for 500 parents to 11.6 min for 100,000 parents, while peak memory usage rose from roughly 0.9–126 GB (Additional File 1, Table 4). For 10-trait indices, runtimes rose from about 0.20–28.1 min over the same range, with peak memory increasing from approximately 1.6–216 GB. Thus, for a conventional 50 k commercial panel applied to the mating-candidate set, multi-trait MSV/MSC computations remain tractable on high-memory evaluation servers, with RAM rather than CPU time becoming the limiting resource at the upper end of this range.

### Benchmarking of the haplotype similarity matrix

We further evaluated *PyMSQ*’s construction of the haplotype-based similarity matrix, an ${\rm O}(N^{2} )$ procedure for $N$ parents (Additional File 1, Table 3). In the Individuals scenario (population sizes of 5000 to 100,000, with 1000 markers), *PyMSQ*’s runtime for building this matrix rose from about 0.08–7.93 min, and peak memory from 0.76 to nearly 70 GB. Conversely, in the Markers scenario (500 individuals, 2500–50,000 markers), similarity matrix runtime grew from 0.04 to 2.8 min, and memory usage climbed from 0.37 to ~ 34.28 GB. These single-chromosome stress tests confirm the expected ${\rm O}(N^{2} )$ scaling of dense similarity matrices and show that large servers can accommodate similarity calculations for either large $N$ or high marker density, thereby enabling refined selection and mating strategies that directly target critical heterozygous segments.

Construction of the aggregate-genotype similarity matrix in the same 50 k-chip setting was substantially more demanding (Additional File 1, Table 5). For single-trait indices, runtimes increased from roughly 0.07 min for 500 parents to 27.3 min for 100,000 parents, while peak memory usage rose from about 1.1–321 GB. For 10-trait indices, the corresponding runtimes ranged from about 0.10 min to 350 min (≈6 h), with peak memory usage increasing from approximately 2.0–507 GB. For the similarity matrix, increasing the number of parents from 10,000 to 100,000 (tenfold) raised runtime by about 46-fold (single trait) and 83-fold (10 traits), and peak memory usage by roughly 16-fold in both cases—substantially steeper than the roughly tenfold increases seen for MSV/MSC in the same 50 k-chip benchmark (Additional File 1, Tables 4, 5). In very large candidate sets, this scaling makes it more practical to restrict similarity calculations to a pre-selected subset of parents and/or to selected chromosomes, rather than to the full population and genome.

### Limitations and future directions

Though *PyMSQ* is both fast and versatile, its in-memory design can be demanding on hardware with limited random access memory (RAM), as reflected in the peak memory values reported in our benchmarks. For MSV/MSC calculations, users with more constrained resources can supply data chromosome by chromosome (or in chromosomal blocks) and run *PyMSQ* sequentially, merging per-chromosome results to substantially reduce peak memory at the cost of longer runtime. For the haplotype-based similarity kernel, the dense $N \times N$ structure makes full matrices impractical for very large parent sets; in such cases, it is natural to restrict similarity calculations to the subset of candidates actually considered for mating and, if needed, to selected chromosomes that are most relevant for the index. Within these bounds, the similarity matrix is most effectively used as a diversity kernel in gain–diversity optimization (e.g., coancestry-based OCS or Mendelian-sampling-based OCS), where upper bounds or penalties on similarity between candidate parents help preserve within-family variance and haplotype diversity in the index of interest. A detailed simulation-based evaluation of such Mendelian-sampling-based OCS schemes, including their impact on long-term genetic gain, inbreeding and haplotype diversity, is given in Musa and Reinsch [14]; here we focus on the computational implementation and refer readers to that paper for breeding-program performance under different selection scenarios. Because the similarity kernel is constructed from phased genotypes, a recombination map, and trait-specific marker-effect estimates, systematic errors in any of these inputs propagate directly into the matrix: inaccurate maps or phasing mainly blur the localization of shared segments, whereas poorly estimated marker effects down-weight truly important regions and up-weight noise, weakening the alignment between similarity constraints and long-term response. *PyMSQ* currently focuses on additive genetic models; adding dominance or epistasis would broaden its scope, while further chunk-based or parallel-streaming optimizations could shrink memory overhead. We emphasize that *PyMSQ* does not replace genomic prediction methods but explicitly serves as a post-processing step applied directly to marker effects derived from genomic prediction methods (Single-Step or Two-Step). *PyMSQ* explicitly calculates MSV, MSC, and haplotype similarity based exclusively on genomic marker-effect estimates. In breeding programs that currently run single-step GBLUP but do not routinely export SNP effects, adopting *PyMSQ* would therefore require an additional SNP-effect extraction step (e.g., back-solving or marker-regression using existing software), rather than any change to the underlying evaluation model. It does not directly incorporate the additional polygenic (residual additive genetic) component that is explicitly modeled using pedigree or genomic relationships in single-step genomic evaluations. Finally, our tests focused on one cattle population; applying *PyMSQ* to other species (swine, poultry, crops) should help confirm its broader applicability for sustaining within-family genetic diversity.

## Conclusions

*PyMSQ* unifies advanced Mendelian (co)variance computation with haplotype-based similarity in a single, open-source platform, enabling faster multi-trait analyses than existing methods. It affords breeders a potent tool to harness within-family variance and safeguard genetic diversity, potentially working in combination with coancestry-based or optimal contribution approaches. Future *PyMSQ* releases will refine data handling, incorporate non-additive genetic models, and explore multi-species applications, ensuring it remains a practical, high-performance resource for modern genomic selection.

## Availability and requirements

Project name: *PyMSQ.*

Project home page: https://github.com/aromemusa/PyMSQ

Operating systems: Platform independent.

Programming language: Python 3.8 + 

Other requirements: *pandas*, *NumPy*, *SciPy*, and *Numba* Python libraries.

License: MIT license.

Any restrictions to use by non-academics: None.

All code, documentation, and the Holstein–Friesian dataset used in the examples are available through the GitHub repository. Users may run *PyMSQ* directly in Python or call it from R via the *reticulate* package. Contributions, including issue reports, feature requests, or new functionalities, are welcome via GitHub’s issue tracker.

## Supplementary information

**Supplementary information** accompanies this paper at https://github.com/aromemusa/PyMSQ/blob/main/paper/Additional_file_1.pdf.

## Supplementary Information

Below is the link to the electronic supplementary material.

Supplementary Material 1

## Data Availability

The code, documentation, illustration, and Holstein–Friesian cattle data are available at https://github.com/aromemusa/PyMSQ.

## Abbreviations

FY: Fat yield (kg)
GBLUP: Genomic best linear unbiased prediction
GEBV: Genomic estimated breeding value
GRM: Genomic relationship matrix
JIT: Just-in-time (compilation)
MSC: Mendelian sampling covariance
MSQ: Mendelian sampling–related quantities
MSV: Mendelian sampling variance
OCS: Optimal contribution selection
pH: Acidity measure (hydrogen-ion concentration)
PY: Protein yield (kg)
RAM: Random access memory
SNP-BLUP: SNP best linear unbiased prediction
