# Supplementary material for: PyMSQ: a Python package for fast Mendelian sampling (co)variance and haplotype-based similarity in genomic selection
Source: BMC Bioinformatics. 2026 Feb 14;27:50. doi: 10.1186/s12859-026-06392-5 (PMC12930755; doi:10.1186/s12859-026-06392-5)
Supplement: Supplementary file 1 — Supplementary Material 1 [file 12859_2026_6392_MOESM1_ESM.pdf]

An R script to generate Figs. 1 - 3 and Performance Comparison Tables

Musa AA and Reinsch N

08-04-2025

```
knitr::opts_chunk$set(echo = TRUE)
options(scipen = 999)  # Prevent scientific notation on axes
```

Overview

This document loads data, processes it, and generates the figures and performance comparison tables referred to in the main manuscript:

1. Figure 1: Density plots of Mendelian sampling variances (MSV) for three milk traits and of the corresponding Mendelian correlations (MSC).
2. Figure 2: Unstandardized and standardized similarity matrices for the aggregate genotype of the three-trait index, with cows grouped by paternal half-sib family.
3. Figure 3: Benchmark plots comparing computation time and memory usage for PyMSQ and gamevar in two single-chromosome stress-test scenarios.
4. Performance Comparison Tables 1 and 2: Summaries of the single-chromosome benchmarks in the Individuals and Markers scenarios, reporting mean runtime (in minutes), peak memory usage (in GB), and time and memory ratios for PyMSQ versus gamevar.
5. Performance Table 3: Benchmark results for PyMSQ's construction of the haplotype-based similarity matrix in the same Individuals and Markers scenarios.
6. Performance Table 4: Runtime and peak memory usage for MSV/MSC in the 50k-chip multi-chromosome benchmark, contrasting single-trait and ten-trait indices across parent-set sizes.
7. Performance Table 5: Runtime and peak memory usage for the aggregate-genotype similarity matrix in the same 50k-chip multi-chromosome benchmark for single-trait and ten-trait indices.

Data and benchmark scenarios

The analyses in this document draw on three related datasets:

1. **Holstein–Friesian example data (bundled with PyMSQ).** This dataset comprises 265 Holstein–Friesian cows from five paternal half-sib families. For each cow, phased genotypes, a physical and approximate genetic map, and marker-effect estimates are available for three milk traits: fat yield (FY), protein yield (PY), and pH. These files (map, phased genotypes, marker effects, and pedigree information) are distributed with the PyMSQ package and provide the basis for the empirical MSV/MSC and similarity-matrix examples.
2. **Single-chromosome benchmark scenarios.** To benchmark PyMSQ against gamevar, we use synthetic single-chromosome datasets under two stress-test scenarios, each analyzed for ten traits:

- **Individuals scenario:** the number of markers on the chromosome is fixed at 1,000, while the number of individuals increases from 5,000 to 100,000 to study scaling in population size.
- **Markers scenario:** the number of individuals is fixed at 500, while the number of SNPs on the chromosome increases from 2,500 to 50,000 to study scaling in marker density.

For each combination of scenario and size, we record computation time (in seconds, later converted to minutes for presentation) and peak memory usage (in GB) for MSV/MSC and for the haplotype-based similarity matrix.

3. **50k-chip multi-chromosome benchmark.** To complement the single-chromosome stress tests with a more conventional whole-genome setting, we construct a benchmark based on a bovine 50k marker map with 39,780 autosomal SNPs distributed over 29 chromosomes. Using this map, we simulate phased haplotypes and marker effects for a single-trait and a ten-trait index and compute MSV/MSC and aggregate-genotype similarity matrices for candidate-parent sets ranging from 500 to 100,000 parents, recording runtime and peak memory usage for each configuration.

1. Density Plots

This section applies PyMSQ to the bundled Holstein–Friesian dataset to compute Mendelian sampling variances and correlations for the three milk traits (fat yield, protein yield, and pH), and generates the density plots shown as Figure 1 in the main manuscript.

Import packages

We load the required libraries

```r
library(ggplot2)      # For creating plots
library(reshape2)     # For reshaping data
library(corrplot)     # For correlation plots
require(RColorBrewer) # For color palettes
library(pheatmap)     # For heatmaps
library(ggpubr)       # For arranging ggplot figures
library(dplyr)         # For data manipulation
library(knitr)        # For document generation
library(reticulate)   # For interfacing with Python
```

Import Data from PyMSQ

```r
# Replace "pymsq_dev" with the conda environment or virtualenv name.
# use_condaenv("pymsq_dev", required = TRUE)
# py_config()  # confirm your environment is active

msq <- import("PyMSQ")
data <- msq$load_package_data()  # now you're set to proceed below
gmap <- data[["chromosome_data"]]        # genetic map
meff <- data[["marker_effect_data"]]     # marker effects
gmat <- data[["genotype_data"]]          # phased genotype
group <- data[["group_data"]]            # group data
ped <- data[["pedigree_data"]]

# define number of traits and index weight
no_traits <- ncol(meff)                 # 3 traits
index_wt <- c(1, 1, 1)
```

Compute Mendelian (co-)variance and correlation using PyMSQ

```r
# Derive population covariance matrix for each chromosome
exp_ldmat <- msq$expldmat(gmap = gmap, group = group, mposunit = "cM")

# Compute Mendelian (co-)variance
msvmsc <- msq$msvarcov(gmat, gmap, meff, exp_ldmat = exp_ldmat, group = group,
                       indwt = index_wt, progress = TRUE)

# Compute Mendelian correlation
mscorr <- msq$msvarcov_corr(msvmsc)
```

Prepare and Plot the Density Data

We extract, reshape, and plot the data:

```r
# Extract relevant columns
msv_traits <- data.frame(msvmsc[, c("fat", "protein", "pH")])

# Compute coefficient of variation for each column
round(sapply(msv_traits, function(x) sd(x) / mean(x)) * 100, 2)

mscorr_traits <- data.frame(mscorr[, c("protein_fat", "pH_fat", "pH_protein")])

# Compute mean correlation
round(sapply(mscorr_traits, function(x) mean(x)), 2)

# Compute range of correlations
round(sapply(mscorr_traits, function(x) range(x)), 3)

# Compute percentage of negative values
round(sapply(mscorr_traits, function(x) (sum(x < 0)/265)*100), 1)

# Reshape data
df_msv <- melt(msv_traits)
colnames(df_msv) <- c("Trait", "Variance")
df_mscorr <- melt(mscorr_traits)
colnames(df_mscorr) <- c("Trait", "Correlation")

# Create density plots
plot1 <- ggplot(df_msv, aes(Variance, color = Trait)) +
  stat_density(geom = "line", position = "identity") +
  theme_classic() +
  theme(legend.position = "top") +
  scale_color_manual(values = c("black", "blue", "red")) +
  ylab("Density")

plot2 <- ggplot(df_mscorr, aes(Correlation, color = Trait)) +
  stat_density(geom = "line", position = "identity") +
  theme_classic() +
  theme(legend.position = "top") +
  scale_color_manual(values = c("black", "blue", "red")) +
  ylab("Density") +
  scale_x_continuous(limits = c(-0.3, 1), breaks = seq(-0.25, 1, by = 0.25))
```

Save and Display Figure 1

We combine the density plots and save them as a TIFF file. We then embed the resulting image:

```
## pdf
##   2
```

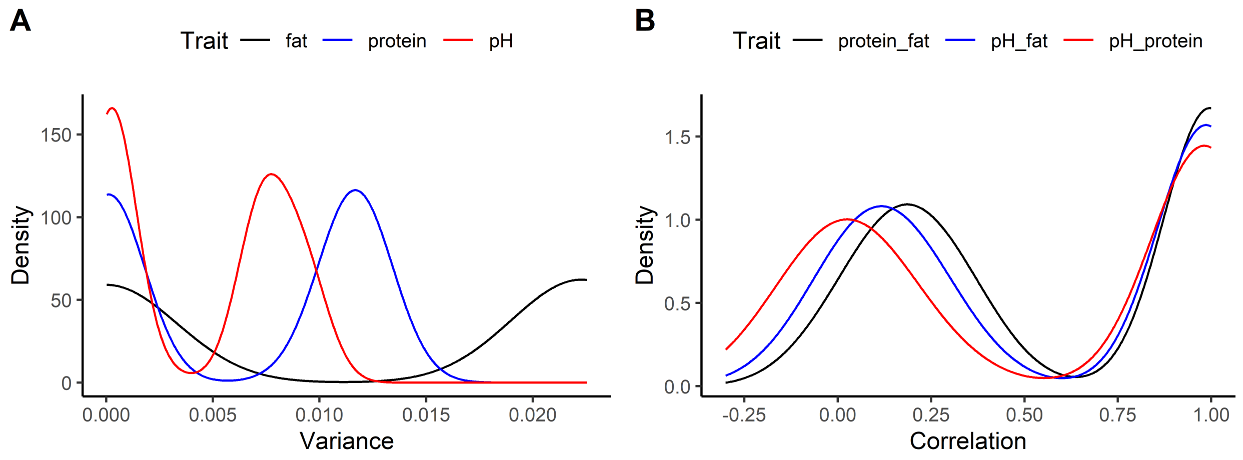

Figure 1: Density plots of Mendelian sampling variances and trait correlations. Panel A displays the variance in fat yield (FY, kg), protein yield (PY, kg), and pH (mol/L). Panel B shows correlations between these traits.

2. Similarity Matrices

Using the same Holstein–Friesian dataset and three-trait index, we next construct a haplotype-based similarity matrix for the aggregate genotype. Diagonal elements correspond to each cow's Mendelian sampling variance, and off-diagonals quantify the shared heterozygous segments between pairs of cows. This section derives the unstandardized and standardized similarity matrices used to produce Figure 2 in the main manuscript.

```
# Derive similarity matrix
sim <- msq$simmat(gmat, gmap, meff, group = group, exp_ldmat = exp_ldmat,
              indwt = index_wt, save=TRUE, progress = TRUE)[[1]]
# Standardize the similarity matrix
std_sim <- cov2cor(sim)

# get the range of values from both matrices
sim_without_diag <- sim
diag(sim_without_diag) <- NA
range(sim_without_diag, na.rm = TRUE)

std_sim_without_diag <- std_sim
diag(std_sim_without_diag) <- NA
range(std_sim_without_diag, na.rm = TRUE)

# find the minimum value in sim and check the corresponding value in std_sim
minVal <- min(sim_without_diag, na.rm = TRUE)
minPos <- which(sim_without_diag == minVal, arr.ind = TRUE)
correspondingPos <- std_sim[minPos[1], minPos[2]]
```

Save and Display Figure 2

```
# Determine number of individuals within each paternal half-sib family
pedigree <- data[["pedigree_data"]][-1, ]
ped <- as.data.frame(pedigree[,2])
no <- NULL
for (i in 1:length(unique(ped[, 1]))) {
  first <- length(which(ped[, 1] == i))
  if (i == 1) {
    no <- c(no, first)
  } else {
    no <- c(no, first + no[i-1])
  }
}

png("Figure2.png", width = 8, height = 4, units = 'in', res = 700)
par(mfrow = c(1, 2), oma = c(0, 0, 0, 0.1) + 0.1, mar = c(0, 0, 0, 0) + 0.1)
cols <- brewer.pal(9, "Blues")
corrplot(sim, is.corr = FALSE, method = "color", cl.lim = range(sim),
         cl.digits = 1, cl.cex = 0.80, tl.col = "black", tl.pos = "n",
         col = cols, cl.align.text = "c", mar = c(0, 0, 1, 0)) -> p
corrRect(p, c(1, no), col = "red")

corrplot(std_sim, is.corr = FALSE, method = "color", cl.lim = range(std_sim),
         cl.digits = 1, cl.cex = 0.80, tl.col = "black", tl.pos = "n",
         col = cols, cl.align.text = "c", mar = c(0, 0, 1, 0)) -> p
corrRect(p, c(1, no), col = "red")

mtext(expression(bold("A")), side = 3, outer = TRUE, cex = 1, las = 0, line = -1, adj = 0)
mtext(expression(bold("B")), at = 0.52, side = 3, outer = TRUE, cex = 1, las = 0, line = -1)

dev.off()
```

```
## pdf
##   2
```

Now we display Figure 2:

3. Benchmark Plots

We then process benchmark data (computation time and memory usage) and generate a 2×2 panel plot.

```
library(dplyr)
library(ggplot2)

# Read csv files
perf_ind <- read.csv("performance_analysis.csv")
perf_mark <- read.csv("performance_analysis_mark.csv")

# Compute computation time (convert seconds to minutes) and its SD
time_ind_summary <- perf_ind %>%
  group_by(no_individuals) %>%
  summarise(
    PyMSQ_mean = mean(time_PyMSQ, na.rm = TRUE) / 60,
    PyMSQ_sd   = sd(time_PyMSQ, na.rm = TRUE) / 60,
```

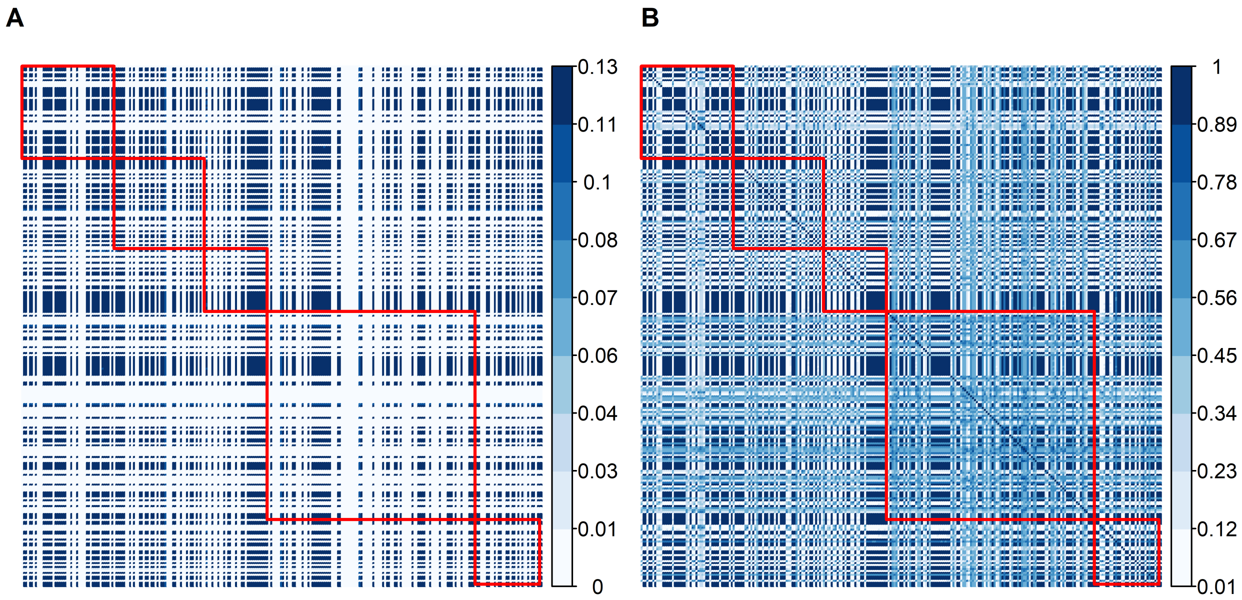

Figure 2: Unstandardized (A) and standardized (B) similarity matrices for the aggregate genotype of some milk traits for 265 cows from five half-sib families (separated by red lines).

```
    gamevar_mean = mean(time_gamevar, na.rm = TRUE) / 60,
    gamevar_sd   = sd(time_gamevar, na.rm = TRUE) / 60
  )

# Compute peak memory usage (GB) and its SD
mem_ind_summary <- perf_ind %>%
  group_by(no_individuals) %>%
  summarise(
    PyMSQ_mean = mean(peak_memory_usage_PyMSQ, na.rm = TRUE),
    PyMSQ_sd   = sd(peak_memory_usage_PyMSQ, na.rm = TRUE),
    gamevar_mean = mean(peak_memory_usage_gamevar, na.rm = TRUE),
    gamevar_sd   = sd(peak_memory_usage_gamevar, na.rm = TRUE)
  )

# Compute computation time (in minutes) & its SD (markers)
time_mark_summary <- perf_mark %>%
  group_by(no_markers) %>%
  summarise(
    PyMSQ_mean = mean(time_PyMSQ, na.rm = TRUE) / 60,
    PyMSQ_sd   = sd(time_PyMSQ, na.rm = TRUE) / 60,
    gamevar_mean = mean(time_gamevar, na.rm = TRUE) / 60,
    gamevar_sd   = sd(time_gamevar, na.rm = TRUE) / 60
  )

# Compute peak memory usage (in GB) & its SD (markers)
mem_mark_summary <- perf_mark %>%
  group_by(no_markers) %>%
  summarise(
    PyMSQ_mean = mean(peak_memory_usage_PyMSQ, na.rm = TRUE),
```

```
    PyMSQ_sd   = sd(peak_memory_usage_PyMSQ, na.rm = TRUE),
    gamevar_mean = mean(peak_memory_usage_gamevar, na.rm = TRUE),
    gamevar_sd   = sd(peak_memory_usage_gamevar, na.rm = TRUE)
  )
```

Save and Display Benchmark Plot (Figure 3)

```
png("Figure3.png", width = 9, height = 8, units = "in", res = 700)
par(mfrow = c(2, 2), oma = c(5,3,1,4.6)+0.1, mar = c(2,1,1,0)+0.1)

# Panel A: Computation Time vs. Individuals
with(time_ind_summary, {
  plot(no_individuals, PyMSQ_mean, type = "l", col = "blue",
       ylim = range(c(PyMSQ_mean - PyMSQ_sd, PyMSQ_mean + PyMSQ_sd,
                      gamevar_mean - gamevar_sd, gamevar_mean + gamevar_sd)),
       xlab = "", ylab = "Time (min)", xaxt = "n", yaxt = "n")
  axis(2, col.axis = "black", las = 2)
  box()
  lines(no_individuals, gamevar_mean, type = "l", col = "red")
  # PyMSQ error bars
  arrows(no_individuals, PyMSQ_mean - PyMSQ_sd,
         no_individuals, PyMSQ_mean + PyMSQ_sd,
         angle = 90, code = 3, col = "blue", length = 0.05)
  # gamevar error bars
  arrows(no_individuals, gamevar_mean - gamevar_sd,
         no_individuals, gamevar_mean + gamevar_sd,
         angle = 90, code = 3, col = "red", length = 0.05)
})
mtext(expression(bold("A")), side = 3, line = 0.5, adj = 0)

# Panel B: Computation Time vs. Markers
with(time_mark_summary, {
  plot(no_markers, PyMSQ_mean, type = "l", col = "blue",
       ylim = range(c(PyMSQ_mean - PyMSQ_sd, PyMSQ_mean + PyMSQ_sd,
                      gamevar_mean - gamevar_sd, gamevar_mean + gamevar_sd)),
       xlab = "Number of Markers", ylab = "", axes = FALSE)
  axis(4, col.axis = "black", las = 2)
  box()
  lines(no_markers, gamevar_mean, type = "l", col = "red")
  arrows(no_markers, PyMSQ_mean - PyMSQ_sd,
         no_markers, PyMSQ_mean + PyMSQ_sd,
         angle = 90, code = 3, col = "blue", length = 0.05)
  arrows(no_markers, gamevar_mean - gamevar_sd,
         no_markers, gamevar_mean + gamevar_sd,
         angle = 90, code = 3, col = "red", length = 0.05)
})
mtext(expression(bold("B")), side = 3, line = 0.5, adj = 0)

# Panel C: Peak Memory Usage vs. Individuals
with(mem_ind_summary, {
  plot(no_individuals, PyMSQ_mean, type = "l", col = "blue",
       ylim = range(c(PyMSQ_mean - PyMSQ_sd, PyMSQ_mean + PyMSQ_sd,
                      gamevar_mean - gamevar_sd, gamevar_mean + gamevar_sd)),
```

```r
       xlab = "Number of Individuals", ylab = "Peak Memory Usage (GB)",
       axes = FALSE)
  axis(2, col.axis = "black", las = 2)
  box()
  lines(no_individuals, gamevar_mean, type = "l", col = "red")
  arrows(no_individuals, PyMSQ_mean - PyMSQ_sd,
         no_individuals, PyMSQ_mean + PyMSQ_sd,
         angle = 90, code = 3, col = "blue", length = 0.05)
  arrows(no_individuals, gamevar_mean - gamevar_sd,
         no_individuals, gamevar_mean + gamevar_sd,
         angle = 90, code = 3, col = "red", length = 0.05)
  axlab = seq(0, max(no_individuals), length.out=11)
  Axis(side = 1, at = axlab, labels = format(axlab, scientific = FALSE), las = 2)
})
mtext(expression(bold("C")), side = 3, line = 0.5, adj = 0)

# Panel D: Peak Memory Usage vs. Markers
with(mem_mark_summary, {
  plot(no_markers, PyMSQ_mean, type = "l", col = "blue",
       ylim = range(c(PyMSQ_mean - PyMSQ_sd, PyMSQ_mean + PyMSQ_sd,
                      gamevar_mean - gamevar_sd, gamevar_mean + gamevar_sd)),
       xlab = "Number of Markers", ylab = "", axes = FALSE)
  axlab = seq(0, max(no_markers), length.out=11)
  Axis(side = 1, at = axlab, labels = axlab, las = 2)
  axis(4, col.axis = "black", las = 2)
  box()
  lines(no_markers, gamevar_mean, type = "l", col = "red")
  arrows(no_markers, PyMSQ_mean - PyMSQ_sd,
         no_markers, PyMSQ_mean + PyMSQ_sd,
         angle = 90, code = 3, col = "blue", length = 0.05)
  arrows(no_markers, gamevar_mean - gamevar_sd,
         no_markers, gamevar_mean + gamevar_sd,
         angle = 90, code = 3, col = "red", length = 0.05)
})
mtext(expression(bold("D")), side = 3, line = 0.5, adj = 0)

# Add global x and y labels
mtext("Time (min)", at = .75, side = 2, outer = TRUE, cex = 1.2, las = 0, line = 1.8)
mtext("Peak memory usage (GB)", at = .25, side = 2, outer = TRUE, cex = 1.2, las = 0, line = 1.8)
mtext("Time (min)", at = .75, side = 4, outer = TRUE, cex = 1.2, las = 0, line = 3.5)
mtext("Peak memory usage (GB)", at = .25, side = 4, outer = TRUE, cex = 1.2, las = 0, line = 3.5)
mtext("Number of individuals", at = .25, side = 1, outer = TRUE, cex = 1.2, las = 0, line = 2)
mtext("Number of markers", at = .75, side = 1, outer = TRUE, cex = 1.2, las = 0, line = 2)

# Common legend
par(fig = c(0, 1, 0, 1), oma = c(0, 0, 0, 0), mar = c(0, 0, 0, 0), new = TRUE)
plot(0, 0, type = 'l', bty = 'n', xaxt = 'n', yaxt = 'n')
legend("bottom", legend = c("gamevar", "PyMSQ"), col = c("red", "blue"),
       lty = c(1, 1), lwd = 1.5, xpd = TRUE, horiz = TRUE, cex = 1.5, seg.len = 1,
       bty = 'n', ncol = 1, y)

dev.off()
```

Now we display Figure 3:

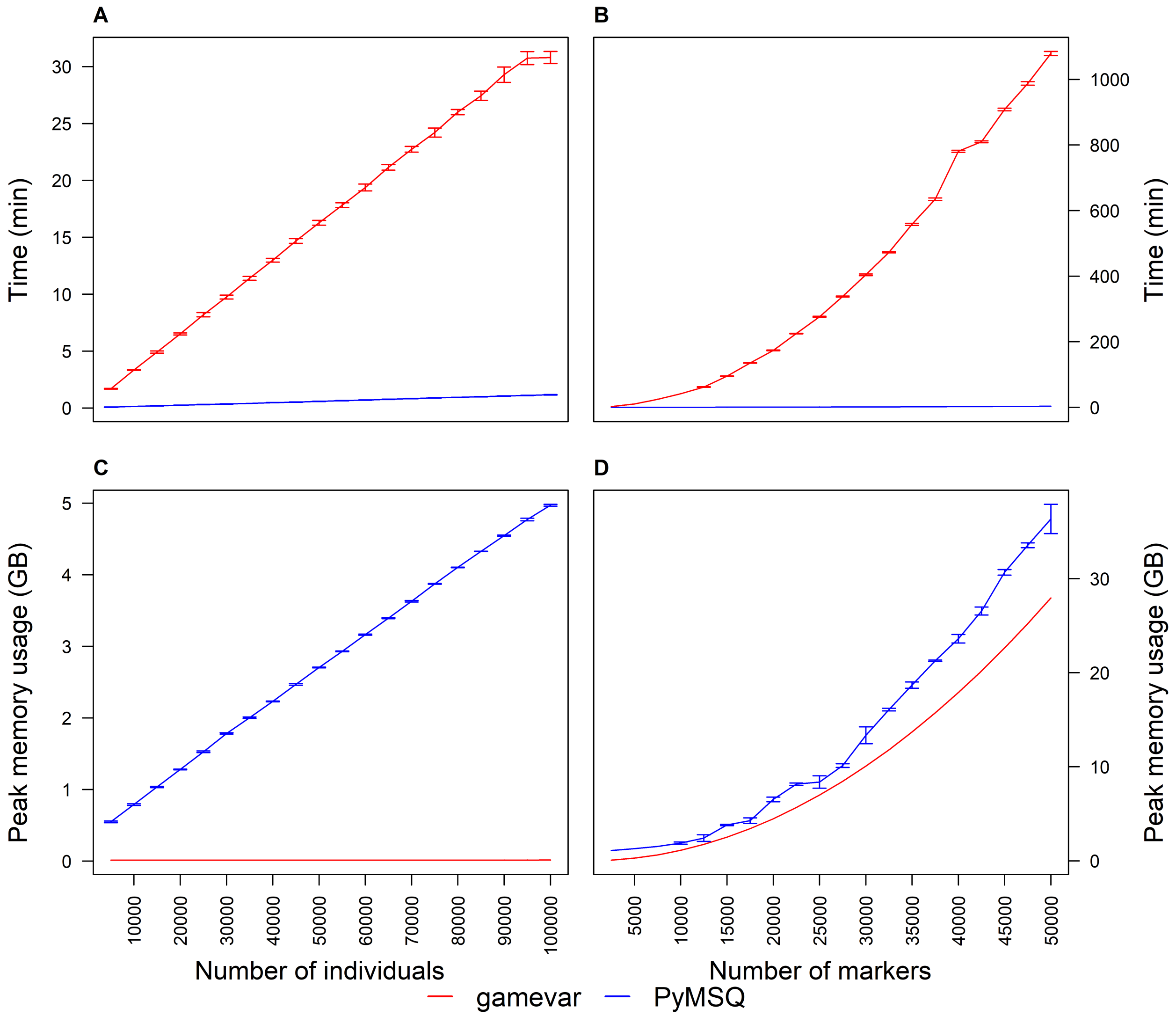

Figure 3: Benchmark plots comparing computation time (Panels A, B) and memory usage (Panels C, D) for PyMSQ and gamevar.

4. Performance Comparison Tables

Using the single-chromosome benchmark scenarios described above, we compare PyMSQ and gamevar in two settings:

- **Individuals scenario:** the number of markers is fixed at 1,000, while the number of individuals varies from 5,000 to 100,000.
- **Markers scenario:** the number of individuals is fixed at 500, while the number of markers varies from 2,500 to 50,000.

Both scenarios involve ten traits on a single chromosome. For each combination, we summarize mean runtime (minutes), peak memory usage (GB), and the corresponding time and memory ratios (gamevar/PyMSQ and PyMSQ/gamevar, respectively), matching the benchmark plots in Figure 3 of the main manuscript.

```r
# For the individuals dataset:
time_ind_summary <- perf_ind %>%
  group_by(no_individuals) %>%
  summarise(PyMSQ_time = mean(time_PyMSQ, na.rm = TRUE) / 60,
            Gamevar_time = mean(time_gamevar, na.rm = TRUE) / 60) %>%
  mutate(Fold_Faster = Gamevar_time / PyMSQ_time)

mem_ind_summary <- perf_ind %>%
  group_by(no_individuals) %>%
  summarise(PyMSQ_memory = mean(peak_memory_usage_PyMSQ, na.rm = TRUE),
            Gamevar_memory = mean(peak_memory_usage_gamevar, na.rm = TRUE)) %>%
  mutate(Memory_Ratio = PyMSQ_memory / Gamevar_memory)

# Merge the summaries for individuals:
individuals_summary <- left_join(time_ind_summary, mem_ind_summary, by = "no_individuals") %>%
  rename(`Number of Individuals` = no_individuals,
         `PyMSQ Time (min)` = PyMSQ_time,
         `Gamevar Time (min)` = Gamevar_time,
         `Time Ratio (Gamevar/PyMSQ)` = Fold_Faster,
         `PyMSQ Memory (GB)` = PyMSQ_memory,
         `Gamevar Memory (GB)` = Gamevar_memory,
         `Memory Ratio (PyMSQ/Gamevar)` = Memory_Ratio)

# For the markers dataset:
time_mark_summary <- perf_mark %>%
  group_by(no_markers) %>%
  summarise(PyMSQ_time = mean(time_PyMSQ, na.rm = TRUE) / 60,
            Gamevar_time = mean(time_gamevar, na.rm = TRUE) / 60) %>%
  mutate(Fold_Faster = Gamevar_time / PyMSQ_time)

mem_mark_summary <- perf_mark %>%
  group_by(no_markers) %>%
  summarise(PyMSQ_memory = mean(peak_memory_usage_PyMSQ, na.rm = TRUE),
            Gamevar_memory = mean(peak_memory_usage_gamevar, na.rm = TRUE)) %>%
  mutate(Memory_Ratio = PyMSQ_memory / Gamevar_memory)

# Merge the summaries for markers:
markers_summary <- left_join(time_mark_summary, mem_mark_summary, by = "no_markers") %>%
  rename(`Number of Markers` = no_markers,
         `PyMSQ Time (min)` = PyMSQ_time,
         `Gamevar Time (min)` = Gamevar_time,
```

```
         `Time Ratio (Gamevar/PyMSQ)` = Fold_Faster,
         `PyMSQ Memory (GB)` = PyMSQ_memory,
         `Gamevar Memory (GB)` = Gamevar_memory,
         `Memory Ratio (PyMSQ/Gamevar)` = Memory_Ratio)
```

Display the table for the individuals dataset:

```
kable(individuals_summary,
      caption = "Performance Comparison (Individuals scenario)", digits = 4)
```

Table 1: Performance Comparison (Individuals scenario)

Number of Individuals	PyMSQ Time (min)	Gamevar Time (min)	Time Ratio (Gamevar/PyMSQ)	PyMSQ Memory (GB)	Gamevar Memory (GB)	Memory Ratio (PyMSQ/Gamevar)
5000	0.0735	1.6917	23.0262	0.5492	0.0139	39.5290
10000	0.1340	3.3415	24.9369	0.7896	0.0140	56.2000
15000	0.1945	4.9152	25.2685	1.0361	0.0141	73.5025
20000	0.2380	6.5075	27.3443	1.2809	0.0141	90.6188
25000	0.3052	8.1996	26.8676	1.5272	0.0142	107.2078
30000	0.3567	9.7491	27.3340	1.7837	0.0143	125.1062
35000	0.4025	11.3988	28.3166	2.0036	0.0143	139.8827
40000	0.4731	12.9819	27.4391	2.2311	0.0143	155.8354
45000	0.5218	14.6783	28.1301	2.4683	0.0144	171.4445
50000	0.5775	16.2707	28.1760	2.7052	0.0144	187.2737
55000	0.6475	17.8198	27.5195	2.9305	0.0145	202.6832
60000	0.6973	19.3777	27.7896	3.1631	0.0145	217.8085
65000	0.7651	21.1444	27.6373	3.3932	0.0145	234.1999
70000	0.8220	22.7347	27.6589	3.6307	0.0146	249.1597
75000	0.8868	24.2053	27.2962	3.8740	0.0145	266.3079
80000	0.9366	26.0018	27.7629	4.1030	0.0146	280.3230
85000	0.9942	27.4408	27.6023	4.3259	0.0147	294.8262
90000	1.0470	29.2895	27.9747	4.5471	0.0147	309.1979
95000	1.1101	30.7444	27.6947	4.7721	0.0147	323.7633
100000	1.1609	30.7995	26.5314	4.9705	0.0148	334.9568

Display the table for the markers dataset:

```
kable(markers_summary, caption = "Performance Comparison (Markers scenario)",
      digits = 4)
```

Table 2: Performance Comparison (Markers scenario)

Number of Markers	PyMSQ Time (min)	Gamevar Time (min)	Time Ratio (Gamevar/PyMSQ)	PyMSQ Memory (GB)	Gamevar Memory (GB)	Memory Ratio (PyMSQ/Gamevar)
2500	0.0333	2.4414	73.2419	1.1052	0.0729	15.1675
5000	0.0668	10.4440	156.3479	1.2909	0.2827	4.5664
7500	0.1107	24.6128	222.4045	1.5237	0.6321	2.4105
10000	0.1708	41.5811	243.4966	1.8872	1.1213	1.6831
12500	0.2675	61.8284	231.1198	2.4206	1.7502	1.3830

Number of Markers	PyMSQ Time (min)	Gamevar Time (min)	Time Ratio (Gamevar/PyMSQ)	PyMSQ Memory (GB)	Gamevar Memory (GB)	Memory Ratio (PyMSQ/Gamevar)
15000	0.3605	95.3007	264.3447	3.8162	2.5188	1.5151
17500	0.4679	135.2333	289.0526	4.2710	3.4270	1.2463
20000	0.5714	173.6894	303.9894	6.5295	4.4750	1.4591
22500	0.7248	224.9720	310.3847	8.1461	5.6627	1.4386
25000	0.9020	276.2836	306.3011	8.3819	6.9901	1.1991
27500	1.0024	338.1668	337.3515	10.1369	8.4571	1.1986
30000	1.2325	404.2205	327.9635	13.3481	10.0639	1.3263
32500	1.3395	472.9189	353.0519	16.0859	11.8104	1.3620
35000	1.6101	558.0030	346.5678	18.6937	13.6965	1.3648
37500	1.8505	634.4724	342.8685	21.2675	15.7224	1.3527
40000	2.1511	781.0272	363.0799	23.6134	17.8880	1.3201
42500	2.3476	809.9011	344.9935	26.5606	20.1932	1.3153
45000	2.6547	908.1433	342.0889	30.6722	22.6381	1.3549
47500	3.0119	987.5281	327.8773	33.5492	25.2228	1.3301
50000	3.2457	1078.8873	332.4034	36.3486	27.9472	1.3006

5. Performance Table 3: Benchmark of similarity matrices

Using the same single-chromosome Individuals and Markers scenarios, we benchmarked PyMSQ's construction of the haplotype-based similarity matrix. For each combination of population size and marker density, we report mean runtime (minutes) and peak memory usage (GB) across replicates, separately for the Individuals and Markers scenarios.

```r
# Read csv files
perf_ind <- read.csv("similarity_unsaved.csv")
perf_mark <- read.csv("similarity_mark_unsaved.csv")

# Compute computation time (in minutes) + SD for individuals
time_ind_summary <- perf_ind %>%
  group_by(no_individuals) %>%
  summarise(times = mean(time, na.rm = TRUE) / 60,
            time_sd = sd(time, na.rm = TRUE) / 60)

mem_ind_summary <- perf_ind %>%
  group_by(no_individuals) %>%
  summarise(memory = mean(peak_memory_usage, na.rm = TRUE),
            memory_sd = sd(peak_memory_usage, na.rm = TRUE))

# Markers scenario
time_mark_summary <- perf_mark %>%
  group_by(no_markers) %>%
  summarise(times = mean(time, na.rm = TRUE) / 60,
            time_sd = sd(time, na.rm = TRUE) / 60)

mem_mark_summary <- perf_mark %>%
  group_by(no_markers) %>%
  summarise(memory = mean(peak_memory_usage, na.rm = TRUE),
            memory_sd = sd(peak_memory_usage, na.rm = TRUE))

# Merge time & memory for Individuals
```

```r
ind_summary <- left_join(time_ind_summary, mem_ind_summary, by = "no_individuals") %>%
  mutate(`Time (min)` = paste0(round(times, 2), " ± ", round(time_sd, 2)),
         `Memory (GB)` = paste0(round(memory, 2), " ± ", round(memory_sd, 2))) %>%
  select(no_individuals, `Time (min)`, `Memory (GB)`)

# Merge time & memory for Markers
mark_summary <- left_join(time_mark_summary, mem_mark_summary, by = "no_markers") %>%
  mutate(`Time (min)` = paste0(round(times, 2), " ± ", round(time_sd, 2)),
         `Memory (GB)` = paste0(round(memory, 2), " ± ", round(memory_sd, 2))) %>%
  select(no_markers, `Time (min)`, `Memory (GB)`)

# Rename to avoid duplicates
ind_summary_renamed <- ind_summary %>%
  rename("No. Individuals" = no_individuals, "Time (min) [Ind]" = "Time (min)",
         "Memory (GB) [Ind]" = "Memory (GB)")

mark_summary_renamed <- mark_summary %>%
  rename("No. Markers" = no_markers, "Time (min) [Mark]" = "Time (min)",
    "Memory (GB) [Mark]" = "Memory (GB)")

# Combine side by side
results <- cbind(ind_summary_renamed, mark_summary_renamed)

# Print the merged table
kable(results, caption = "Benchmark results for similarity matrices in the
      Individuals and Markers scenarios")
```

Table 3: Benchmark results for similarity matrices in the Individuals and Markers scenarios

No. Individuals	Time (min) [Ind]	Memory (GB) [Ind]	No. Markers	Time (min) [Mark]	Memory (GB) [Mark]
5000	0.08 ± 0.02	0.76 ± 0.05	2500	0.04 ± 0.01	0.37 ± 0.03
10000	0.17 ± 0.01	1.36 ± 0.04	5000	0.07 ± 0	0.63 ± 0.03
15000	0.3 ± 0	2.33 ± 0.16	7500	0.1 ± 0	1.04 ± 0.06
20000	0.44 ± 0	3.33 ± 0.17	10000	0.17 ± 0	1.57 ± 0.19
25000	0.66 ± 0.02	4.93 ± 0.32	12500	0.23 ± 0	2.33 ± 0.06
30000	0.89 ± 0.02	6.78 ± 0.32	15000	0.34 ± 0	3.15 ± 0.05
35000	1.16 ± 0.02	9.12 ± 0.56	17500	0.4 ± 0.01	4.16 ± 0.04
40000	1.45 ± 0.01	11.71 ± 0.77	20000	0.53 ± 0	5.93 ± 0.22
45000	1.82 ± 0.02	14.32 ± 0.62	22500	0.64 ± 0	7.66 ± 0.12
50000	2.2 ± 0	16.76 ± 0.29	25000	0.79 ± 0.02	7.92 ± 0.03
55000	2.62 ± 0	21.18 ± 0.58	27500	0.94 ± 0	9.45 ± 0.04
60000	3.06 ± 0.02	25.8 ± 0.82	30000	1.07 ± 0	12.24 ± 0.41
65000	3.56 ± 0	29.27 ± 0.9	32500	1.24 ± 0.01	15.05 ± 0.36
70000	4.07 ± 0.02	33.67 ± 0.43	35000	1.44 ± 0	17.57 ± 0.28
75000	4.6 ± 0.03	37.55 ± 0.64	37500	1.67 ± 0.1	20.09 ± 0.19
80000	5.26 ± 0.03	44.73 ± 1.62	40000	1.89 ± 0.08	22.5 ± 0.38
85000	5.86 ± 0.05	50.46 ± 0.62	42500	2.07 ± 0.06	24.78 ± 0.68
90000	6.48 ± 0.05	55.42 ± 0.52	45000	2.32 ± 0.07	30.03 ± 0.43
95000	7.15 ± 0.07	60.44 ± 0.67	47500	2.54 ± 0	32.82 ± 0.35
100000	7.93 ± 0.09	69.58 ± 1.87	50000	2.8 ± 0.02	34.28 ± 0.22

6. Performance Table 4: MSV/MSC on a 50k-chip multi-chromosome panel

Using the 50k-chip multi-chromosome benchmark described in the main manuscript, we evaluated PyMSQ runtimes and peak memory usage for computing MSV/MSC for an aggregate index. Candidate-parent sets ranged from 500 to 100,000 parents, and we contrasted a single-trait index with a ten-trait index. The table below summarizes computation time (in minutes) and peak memory usage (in GB) for each parent-set size.

```r
# Read MSV/MSC benchmark results (50k chip, whole genome)
msv_1  <- read.csv("MSV_MSC_50kchip_1_trait.csv")
msv_10 <- read.csv("MSV_MSC_50kchip_10_trait.csv")

# Prepare single-trait summary: convert time to minutes; memory already in GB
msv_1_sum <- msv_1 %>%
  mutate(`Time (min) [1 trait]`  = round(time / 60, 2),
         `Memory (GB) [1 trait]` = round(peak_memory_usage, 2)) %>%
  select(no_individuals, `Time (min) [1 trait]`, `Memory (GB) [1 trait]`) %>%
  rename(`No. Parents` = no_individuals)

# Prepare ten-trait summary: convert time to minutes; memory already in GB
msv_10_sum <- msv_10 %>%
  mutate(`Time (min) [10 traits]`  = round(time / 60, 2),
         `Memory (GB) [10 traits]` = round(peak_memory_usage, 2)) %>%
  select(no_individuals,`Time (min) [10 traits]`, `Memory (GB) [10 traits]`) %>%
  rename(`No. Parents` = no_individuals)

# Combine 1-trait and 10-trait summaries side by side
table4 <- left_join(msv_1_sum, msv_10_sum, by = "No. Parents")

kable(table4, caption = "Runtime and peak memory usage for PyMSQ when computing
MSV/MSC for single-trait and ten-trait indices in the 50k-chip multi-chromosome
benchmark.")
```

Table 4: Runtime and peak memory usage for PyMSQ when computing MSV/MSC for single-trait and ten-trait indices in the 50k-chip multi-chromosome benchmark.

No. Parents	Time (min) [1 trait]	Memory (GB) [1 trait]	Time (min) [10 traits]	Memory (GB) [10 traits]
500	0.13	0.87	0.20	1.60
1000	0.17	1.28	0.28	2.68
5000	0.63	6.76	1.28	11.27
10000	1.25	13.06	2.63	22.02
20000	2.44	25.66	5.20	43.51
30000	3.60	38.26	8.08	65.00
40000	4.80	50.85	10.62	86.50
50000	5.92	63.45	13.46	107.99
60000	6.85	76.05	16.46	129.48
70000	8.21	88.65	18.71	150.99
80000	9.34	101.24	22.24	172.48
90000	10.39	113.84	27.53	193.97
100000	11.58	126.44	28.10	215.48

7. Performance Table 5: similarity matrices on a 50k-chip multi-chromosome panel

In the same 50k-chip setting, we benchmarked construction of the dense aggregate-genotype similarity matrix for the same series of parent-set sizes. As for MSV/MSC, we contrasted a single-trait index with a ten-trait index. Because the similarity matrix scales quadratically with the number of parents, this benchmark highlights the steeper increase in computational demands, particularly for the ten-trait index. The table summarizes runtime (minutes) and peak memory usage (GB) for each parent-set size.

```r
# Read similarity-matrix benchmark results (50k chip, whole genome)
sim_1  <- read.csv("similarity_matrix_50kchip_1_trait.csv")
sim_10 <- read.csv("similarity_matrix_50kchip_10_traits.csv.csv")

# Prepare single-trait summary: time to minutes; memory already in GB
sim_1_sum <- sim_1 %>%
mutate(`Time (min) [1 trait]`  = round(time / 60, 2),
       `Memory (GB) [1 trait]` = round(peak_memory_usage, 2)) %>%
  select(no_individuals, `Time (min) [1 trait]`, `Memory (GB) [1 trait]`) %>%
  rename(`No. Parents` = no_individuals)

# Prepare ten-trait summary: time to minutes; memory already in GB
sim_10_sum <- sim_10 %>%
  mutate(`Time (min) [10 traits]`  = round(time / 60, 2),
         `Memory (GB) [10 traits]` = round(peak_memory_usage, 2)) %>%
  select(no_individuals, `Time (min) [10 traits]`, `Memory (GB) [10 traits]`) %>%
  rename(`No. Parents` = no_individuals)

# Combine 1-trait and 10-trait summaries side by side
table5 <- left_join(sim_1_sum, sim_10_sum, by = "No. Parents")
kable(table5, caption = "Runtime and peak memory usage for PyMSQ when
      constructing the aggregate-genotype similarity matrix for single-trait
      and ten-trait indices in the 50k-chip multi-chromosome benchmark.")
```

Table 5: Runtime and peak memory usage for PyMSQ when constructing the aggregate-genotype similarity matrix for single-trait and ten-trait indices in the 50k-chip multi-chromosome benchmark.

No. Parents	Time (min) [1 trait]	Memory (GB) [1 trait]	Time (min) [10 traits]	Memory (GB) [10 traits]
500	0.07	1.07	0.10	1.90
1000	0.07	2.38	0.17	3.35
5000	0.27	9.80	1.36	15.33
10000	0.59	19.20	4.22	31.37
20000	1.57	38.01	15.37	66.07
30000	3.25	57.72	33.66	105.61
40000	4.98	84.27	56.42	149.63
50000	7.41	114.61	87.82	198.20
60000	10.61	148.09	125.23	251.02
70000	13.86	186.01	176.33	308.21
80000	17.91	227.42	262.63	370.25
90000	23.44	272.67	281.62	436.15
100000	27.27	321.29	349.91	507.37
